# IFITM3 promotes malignant progression, cancer stemness and chemoresistance of gastric cancer by targeting MET/AKT/FOXO3/c-MYC axis

**DOI:** 10.1186/s13578-022-00858-8

**Published:** 2022-08-08

**Authors:** Pei-Yi Chu, Wei-Chieh Huang, Shiao-Lin Tung, Chung-Ying Tsai, Chih Jung Chen, Yu-Chin Liu, Chia-Wen Lee, Yang-Hsiang Lin, Hung-Yu Lin, Cheng-Yi Chen, Chau-Ting Yeh, Kwang-Huei Lin, Hsiang-Cheng Chi

**Affiliations:** 1 Department of Post-Baccalaureate Medicine, College of Medicine, National Chung Hsing University, Taichung, 402 Taiwan; 2 Department of Pathology, Show Chwan Memorial Hospital, Changhua, 500 Taiwan; 3 School of Medicine, College of Medicine, Fu Jen Catholic University, New Taipei City, 242 Taiwan; 4 Department of Health Food, Chung Chou University of Science and Technology, Changhua, 510 Taiwan; 5 National Institute of Cancer Research, National Health Research Institutes, Tainan, 704 Taiwan; 6 Graduate Institute of Integrated Medicine, China Medical University, Taichung, Taiwan; 7 Chinese Medicine Research Center, China Medical University, Taichung, Taiwan; 8 Department of Hematology and Oncology, Ton-Yen General Hospital, Hsinchu County, Taiwan; 9 Department of Nursing, Hsin Sheng Junior College of Medical Care and Management, Taoyuan City, Taiwan; 10 Kidney Research Center, Department of Nephrology, Chang Gung Memorial Hospital, Taoyuan, Taiwan; 11 Department of Pathology and Laboratory Medicine, Taichung Veterans General Hospital, Taichung, Taiwan; 12 School of Medicine, Chung Shan Medical University, Taichung, Taiwan; 13 Molecular Medicine Research Center, Chang Gung University, Taoyuan, Taiwan; 14 Department of Cell and Molecular Biology, College of Medicine, Chang Gung University, Taoyuan, Taiwan; 15 Department of Ophthalmology, Chang Gung Memorial Hospital, Linkou, Taiwan; 16 Liver Research Center, Chang Gung Memorial Hospital, Linkou, Taoyuan Taiwan; 17 Research Assistant Center, Show Chwan Memorial Hospital, Changhua, 500 Taiwan; 18 Department of Cell Biology and Anatomy, College of Medicine, National Cheng Kung University, Tainan, 70101 Taiwan; 19 Department of Biochemistry, College of Medicine, Chang-Gung University, 259 Wen-hwa 1 Road, Taoyuan, Taiwan; 20 Graduate Institute of Biomedical Sciences, College of Medicine, Chang-Gung University, Taoyuan, Taiwan; 21 Research Center for Chinese Herbal Medicine, College of Human Ecology, Chang Gung University of Science and Technology, Taoyuan, Taiwan

**Keywords:** IFITM3, Gastric cancer, iTRAQ quantitative proteomic analysis, Cancer stemness, MET, FOXO3, c-MYC

## Abstract

**Background:**

Targeting the HGF/MET signaling pathway has been a viable therapeutic strategy for various cancer types due to hyperactivation of HGF/MET axis occurs frequently that leads to detrimental cancer progression and recurrence. Deciphering novel molecule mechanisms underlying complex HGF/MET signaling network is therefore critical to development of effective therapeutics for treating MET-dependent malignancies.

**Results:**

Using isobaric mass tag-based quantitative proteomics approach, we identified IFITM3, an interferon-induced transmembrane protein that was highly expressed in micro-dissected gastric cancer (GC) tumor regions relative to adjacent non-tumor epithelia. Analyses of GC clinical specimens revealed that expression IFITM3 was closely correlated to advanced pathological stages. IFITM3 has been reported as a PIP3 scaffold protein that promotes PI3K signaling. In present study, we unprecedentedly unraveled that IFITM3 associated with MET and AKT to facilitate HGF/MET mediated AKT signaling crosstalk in suppressing FOXO3, consequently leading to c-MYC mediated GC progression. In addition, gene ontology analyses of the clinical GC cohort revealed significant correlation between IFITM3-associated genes and targets of c-MYC, which is a crucial downstream effector of HGF/MET pathway in cancer progression. Moreover, we demonstrated ectopic expression of IFITM3 suppressed FOXO3 expression, consequently led to c-MYC induction to promote tumor growth, cell metastasis, cancer stemness as well as chemoresistance. Conversely, depletion of IFITM3 resulted in suppression of HGF triggered cellular growth and migration via inhibition of AKT/c-MYC signaling in GC.

**Conclusions:**

In summary, our present study unveiled a novel regulatory mechanism for c-MYC-driven oncogenesis underlined by IFITM3-mediated signaling crosstalk between MET associated AKT signaling cascade.

**Supplementary Information:**

The online version contains supplementary material available at 10.1186/s13578-022-00858-8.

## Background

Gastric cancer (GC) ranks the fifth most common cancer and the third most common cause of cancer-related mortality worldwide [1]. Although early stage GC patients feasible for curative surgery retain 70 to 95% 5-year overall survival (OS) rate, more than two thirds of GC patients are diagnosed with advanced stages with unresectable diseases [2, 3]. For unresectable advanced or metastatic GC, the prognosis remains poor with median OS around 8 to 12 months after 1st-line chemotherapy and a 5-year OS at around 5% [4]. Since chemotherapy has been the major treatment option for advanced GC, development of chemoresistance has largely limited the effectiveness of chemotherapy and resulted in disease recurrence and grave prognosis [2]. The mechanisms of chemoresistance in GC are multifactorial that involve in drug efflux, drug interaction and dysregulation of cellular signaling as well as pathways that regulate cancer stemness [2, 5]. Hence, there has been an urgent need to explore new therapeutic targets to overcome chemoresistance in GC.

MET (Mesenchymal-epithelial transition factor) is a receptor tyrosine kinase that is proposed as a promising target in cancer therapy due to the predominant oncogenic signaling cascades that has been shown crucial to malignant progression and tumorigenesis of several cancer types [6–8]. Binding of HGF (Hepatocyte growth factor), for instance, triggers receptor homodimerization and phosphorylation of MET on tyrosine residues, recruiting adaptor and effector proteins that consequently stimulate downstream signaling pathways such as PI3K/AKT, Ras/MAPK and Wnt/β-catenin [8]. In GC, MET gene amplification and overexpression have been reported to correlate with cancer progression and poor prognosis [9, 10]. Hyperactivation of HGF/MET signaling is shown to initiate epithelial–mesenchymal transition (EMT) phenomenon to promote metastasis via enriching cancer stem cells (CSCs) that contribute to chemoresistance [11, 12]. Since CSCs frequently constitute small subpopulation of self-renewal and differentiation properties that lead to tumor formation, drug resistance, metastasis and recurrent diseases [13–15]. Thus, identifying a novel mechanism by which HGF/MET signaling network regulates can be beneficial for strategizing an effective therapeutic regimen.

IFITM3 (Interferon-induced transmembrane protein 3), is a 10–15 kDa protein that belongs to the interferon-inducible transmembrane (IFITM) protein family [16, 17]. IFITM proteins are powerful suppressors of viral infections and play key roles in immune cell signaling, cell adhesion, and stem cell migration [18, 19]. IFITM3 is increasingly reported as a oncogene in various cancer types such as hepatocellular carcinoma (HCC), glioma, B cell malignancies, colon, prostate, breast and gastric cancers [18, 20–25]. Nevertheless, definite regulatory mechanisms underlined by IFTIM3 in promoting gastric cancer progression, cancer stemness and chemoresistance still remain obscure. In present study, we reported that IFITM3 served as a novel modulator of HGF/MET-mediated AKT signaling that suppressed FOXO3 (Forkhead Box O3), consequently leading to c-MYC (MYC proto-oncogene) mediated cancer proliferation, migration, stemness and drug resistance. Targeting IFITM3 may thus concomitantly interfere with the crosstalk between two major signaling pathways and can serve as a promising treatment strategy against malignant GC progression.

## Results

### Overexpression of IFITM3 was found in GC tissues and correlates with advanced stages

**Fig. 1:**
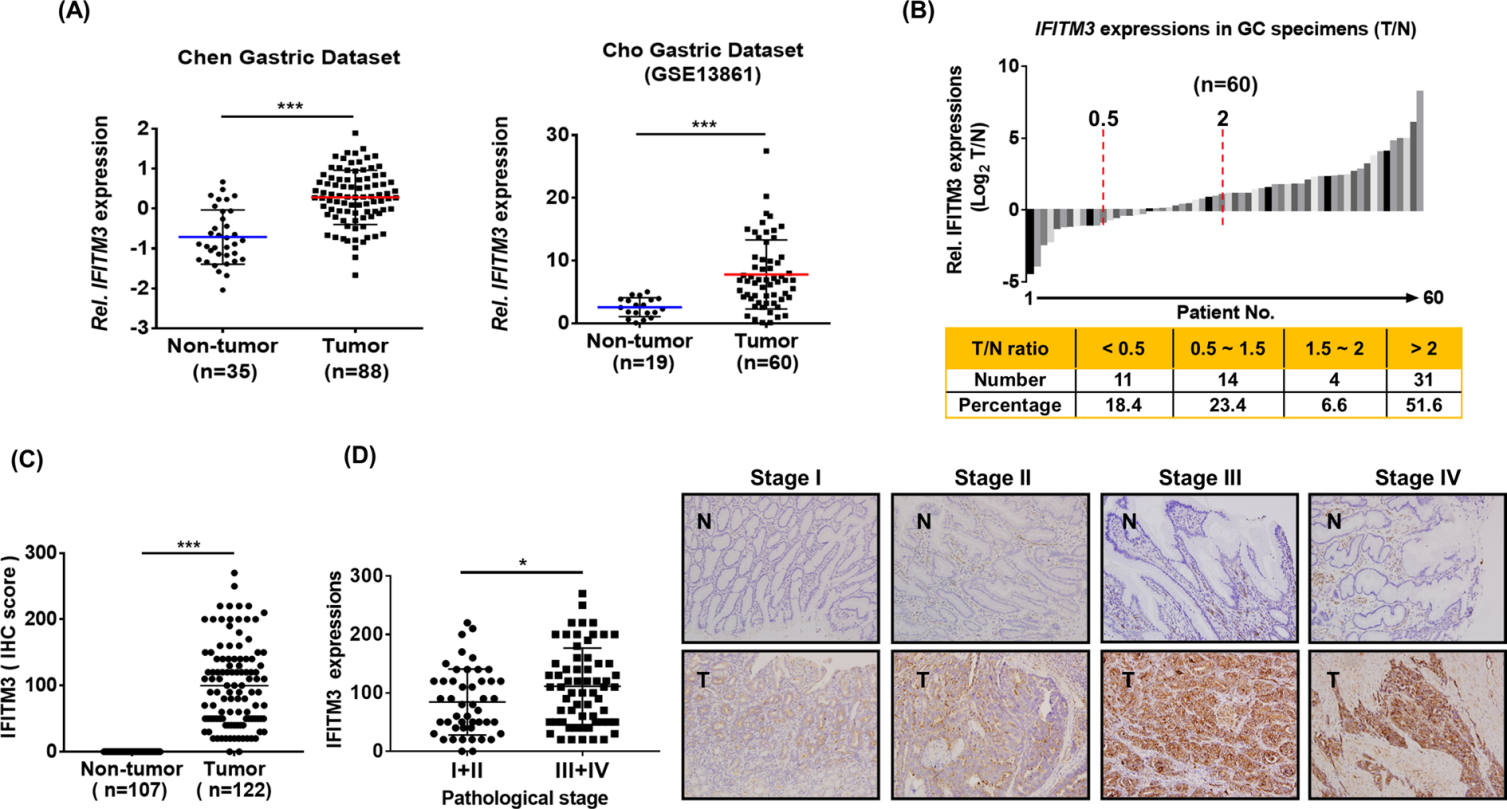
IFITM3 is overexpressed in human gastric cancer and is highly correlated to advanced pathological stages. **A** *IFITM3* expression was analyzed by classification of non-tumor or tumor groups using the Chen and Cho gastric cancer datasets. **B** *IFITM3* expressions in 60 clinical GC specimens were evaluated by RT-qPCR. T/N ratio less than 0.5 was defined as down-regulation, while T/N ratio greater than 2 was defined as up-regulation of *IFITM3*. **C** IHC analysis of IFITM3 in 122 GC tumor tissues (T) and 107 noncancerous adjacent mucosa (N). The y-axis represents IHC score. ***p < 0.001. **D** Scatter plot shows IFITM3 expression in relation to different pathology stages of GC specimens. Right panel displays four representative pairs of GC specimens at different pathological stages from the IHC analyses

To identify novel prognostic factors of GC, we first examined the correlation between 415 overexpressed proteins in GC tumors from our iTRAQ quantitative proteomic database (Additional file 2: Table S1 and Additional file 3: Table S2) and 4377 upregulated transcriptomes in tumors from the Chen dataset [26]. As shown in Fig. 1A, IFITM3 was highly expressed in GC tumors when compared to non-tumor counterparts. Overexpression of IFITM3 in GC cancers was also observed in another independent cohort of patients (Cho, GSE13861)[27] as relative expression of IFITM3 was significantly higher in micro-dissected GC tissues as compared to adjacent normal gastric tissues (Fig. 1A). To consolidate the finding of highly enhanced IFITM3 expression in GC, real-time quantitative polymerase chain reaction (qPCR) was performed on clinical specimens from 60 GC patients. In line with proteomic analyses, our data consistently showed that higher expression of IFITM3 were detected at both mRNA and protein levels. For instance, 58.2% of GC specimens analyzed demonstrated significantly elevated *IFITM3* expression when of T/N ratio was greater 1.5 (Fig. 1B). To further correlate clinical significance of IFITM3, we carried out immunohistochemistry (IHC) staining analyses on tumor (n = 122) and non-tumor (n = 107) regions from the GC specimens. The results showed significantly higher IFITM3 expression in the tumor parts than the non-tumor regions (Fig. 1C). Furthermore, we systemically explored whether IFIMT3 expression could be differentially expressed at different pathological stages in GC. Our data revealed that GC patient specimens at stage III/IV showed greater expression of IFMT3 than those at stage I/II (Fig. 1D). These findings thus indicated that IFITM3 is a novel prognostic molecule that is significantly upregulated in GC tumor, and is closely correlated to GC progression at advanced pathological stages.

### Silencing of IFITM3 inhibits colony formation, cell proliferation, cell migration and chemoresistant properties of GC cells

**Fig. 2:**
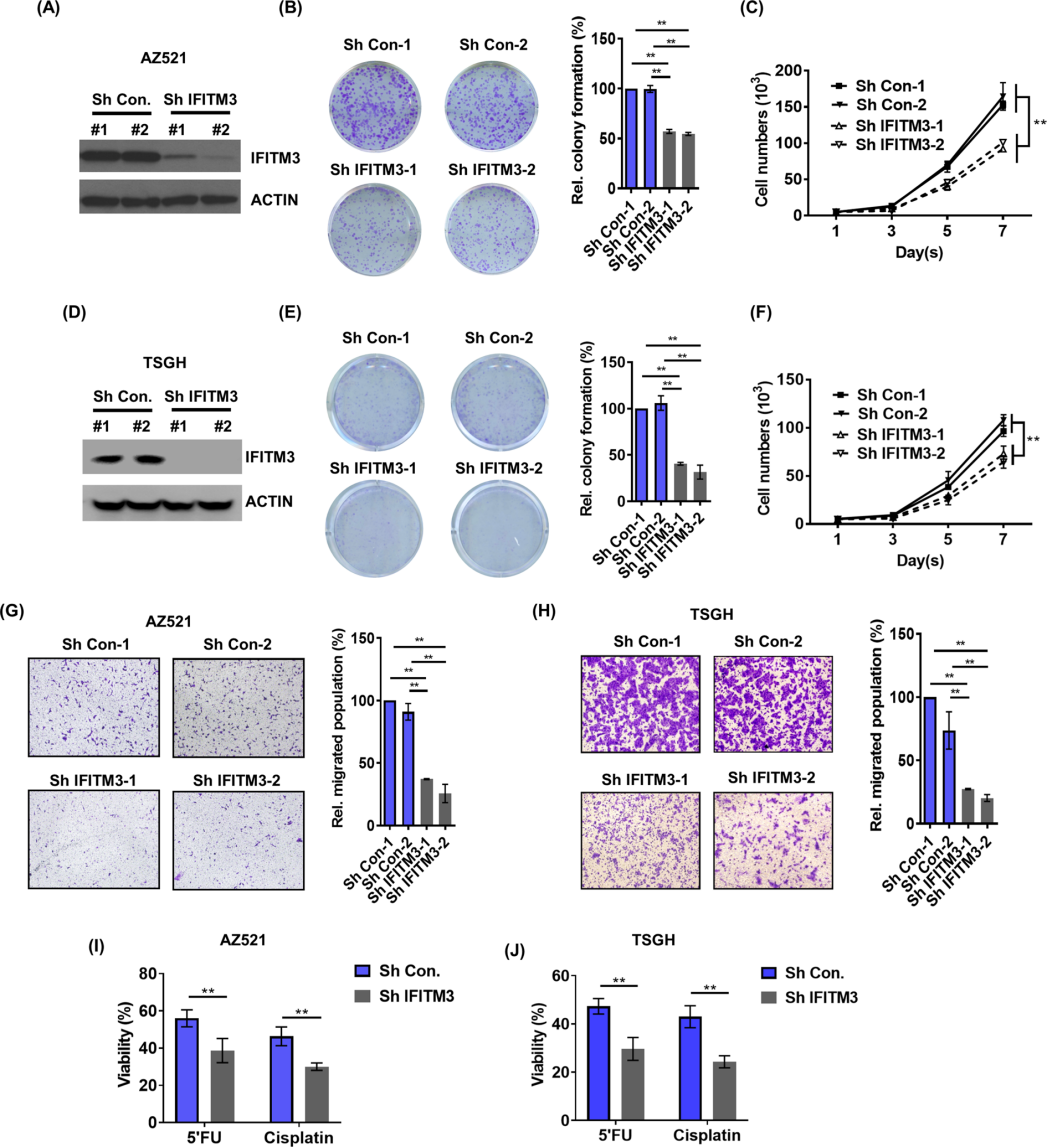
IFITM3 depletion suppresses colony-formation, proliferation, metastasis and chemoresistance properties of GC cells. **A**, **D**) The efficiency of IFITM3 silencing in AZ521 and TSGH cells was determined by western blots. **B**, **E** The left panels show a representative image of the colony formation assay from each treatment group from crystal violet staining. The right panel are the quantitative analyses of the colony formation assays conducted (**p < 0.01). **C**, **F** Cellular viability of the two GC cell lines that had been infected with *IFITM3* shRNAs were analyzed by viable cell counts (**p < 0.01). **G**, **H** Transwell migration assays of *IFITM3*-silenced AZ521 and TSGH cells, respectively. Left panels are representative transwell migration staining images from each group; the right panels show the quantitative analyses on migrated cells (**p < 0.01). **I**, **J** Chemosensitivities of 5’FU or cisplatin-treated AZ521 and TSGH cells, respectively, that were shCon- or shIFITM3-infected. The cellular viabilities (%) were determined based on the relative numbers of viable cells after treatment with 5’FU (20 μg/ml) or cisplatin (3 μM) for 72 h using MTS assay

To assess functional roles of IFITM3 in GC cell lines in vitro, protein expression of IFITM3 was suppressed by knocking down IFITM3 with two IFITM3-specific short hairpin RNAs (shRNAs) in two GC cell lines AZ521 and TSGH (Fig. 2A, D, respectively). Firstly, suppression of IFITM3 by either shRNA clone was found to significantly reduce colony-forming abilities of both AZ521 and TSGH cells (Fig. 2B, E, respectively). Cell proliferation was also greatly impaired by depletion of IFITM3 as compared to that of control cells in both AZ521 and TSGH cells (Fig. 2C, F, respectively). Since silencing IFITM3 demonstrated significant suppression effects on cell viability and colony formation of two GC cell lines, we next examined whether IFITM3 could play a role in regulating cellular migration of GC cells. In our transwell migration assays, the migratory ability of AZ521 cells were greatly reduced when compared to the control groups (Fig. 2G). Similarly, IFITM3-mediated migration was also compromised in shIFITM3 but not control cells of TSGH (Fig. 2H). Since 5-fluorouracil (5’FU) and cisplatin have become a global standard regimen for first-line treatment of metastatic GC [28], we next examined whether IFITM3 contributes to chemoresistant property of GC cells attributed by treatments of 5’FU and cisplatin. Our results revealed that inhibition of IFITM3 by shRNAs significantly rendered both AZ521 and TSGH cells more chemosensitive towards 5’FU and cisplatin (Fig. 2I, J, respectively). Taken together, these findings suggested that IFITM3 is a novel transmembrane protein that plays critical roles in mediating GC progression via regulating colony formation, cell proliferation, cell migration and chemoresistance.

### Overexpressing IFITM3 enhances cancer progression properties and in vivo pulmonary metastasis

**Fig. 3:**
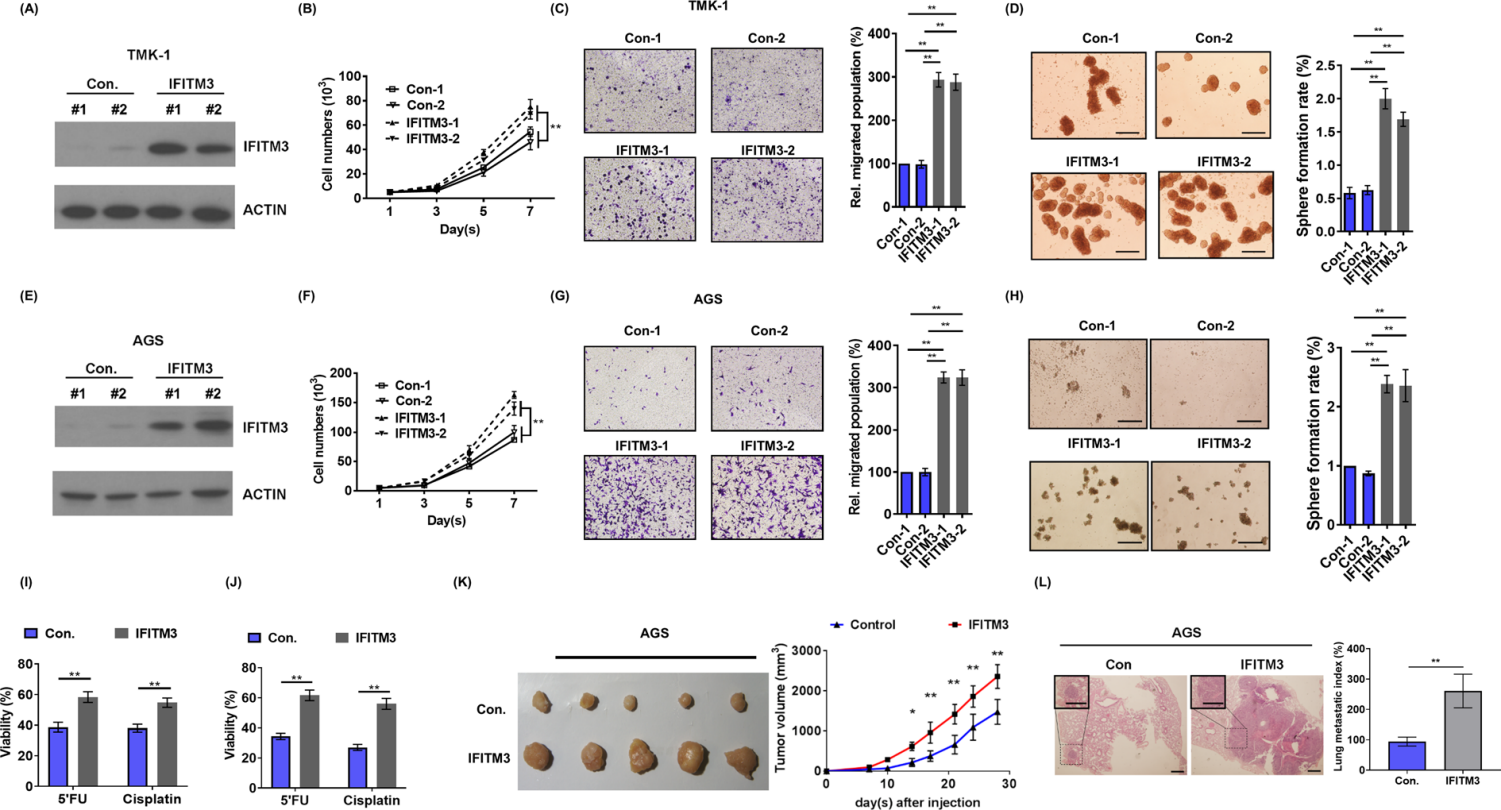
IFITM3 overexpression is central to cancer stemness, cancer progression of GC and pulmonary metastasis in vivo. **A**, **E** The efficiencies of IFITM3 overexpression in TMK-1 and AGS cells were determined by analyzing IFITM3 protein expression using western blots. **B**, **F** Measurements of cellular growth curve by viable cell counts from control or IFITM3-overexpressing TMK-1 and AGS cells, respectively. **C**, **G** Transwell migration assays were conducted using control or IFITM3-overexpressing and TMK-1 and AGS cells, respectively. Left panels: representative images of migrated cells from each treatement group. Right panel: quantitative and statistical analyses (**p < 0.01). **D**, **H** Sphere formation assays were utilized to assess impacts of IFITM3 overexpression on sphere-forming capabilities of TMK-1 and AGS cells (Scale bar, 500 μm). Left panels: representative spheres formed on TMK-1 or AGS1 cells. Right panels: quantitative and statistical analyses (**p < 0.01). **I**, **J** Chemoresistance of control- or IFITM3-overexpressing TMK-1 and AGS cells was determined based on percentages of surviving cells after treatment with 5’FU (20 μg/ml) or cisplatin (3 μM) for 72 h. Viable cells after treatments were measured using MTS assay. **K** Control or IFITM3-overexpessing AGS cells (2 × 10^6^) were subcutaneously injected into nude mice for monitoring tumor growth. Tumors from the tumor-bearing mice (n = 6 for each group) were collected as shown in the left panel image. Tumor growth curves were plotted and statistical differences between control and IFITM3-oeverexpression groups were derived using Student’s *t*-test (**p < 0.01). (L) SCID mice that received intravenous injection of 1 × 10^6^ control- or IFITM3-overexpressing AGS cells (n = 6 per group) were used to investigate if IFITM3 contributed to metastasis. All animals were sacrificed 4 weeks after cancer cell injections, and lungs from control or IFITM3-overexpresing groups were removed for tumor biopsy. Left panel: Representative H&E staining images from lungs of the control and IFITM3 groups of mice. Scale bar: 200 μm. Right panel represents the lung metastatic index (average fold increases in the density of tumor foci as per cm^2^ of lung area)

Our analyses in Fig. 1 implicated IFITM3 is an oncoprotein that is overexpressed in GC specimens at advanced stages. To unravel whether IFITM3 overexpression is indeed associated with progression of GC, we established stable IFITM3-overexpressing cell lines from TMK-1 and AGS (Fig. 3A, E, respectively). In IFITM3-overexpressing TMK-1 and AGS cells, cellular proliferation was significantly increased when compared to the control cells (Fig. 3B, F, respectively). Cell migratory ability was also augmented markedly by IFITM3 overexpression in both cell lines as compared to the control cells (Fig. 3C, G). In order to explore the impact of IFITM3 on cancer stemness, we then enriched CSCs from both TMK-1 and AGS stable cell lines overexpressing IFITM3 in stem-cell-selective conditions [29]. Our results demonstrated that overexpression of IFITM3 greatly enhanced the sphere-forming ability of both TMK-1 and AGS cells as the number of spheres formed was significantly increased when IFITM3 was overexpressed (Fig. 3D, H). Next, we evaluate whether the IFITM3-depletion associated chemosensitivity of AZ521 and TSGH cells could be recapitulated in TMK-1 and AGS IFITM3-overexpression models. The data derived from 5’FU or cisplatin-treatments showed that increased expression of IFITM3 rendered TMK-1 and AGS cells more chemoresistant to 5’FU and cisplatin as significantly more viable cells were recorded in the IFITM3-overexpressing groups (Fig. 3I, J). The above reverse genetics data prompted us next to establish xenograft models and evaluate whether IFITM3-mediated cancer progression properties could be translational in vivo. As shown in Fig. 3K, overexpression of IFITM3 significantly augmented tumor growth of AGS xenografts in *vivo*, supporting the oncogenic property of IFITM3 in GC tumorigenicity. Furthermore, since silencing and overexpressing of IFITM3 had led to compromised and elevated cellular migration, respectively, we also examined if these migratory effects could also be observed in vivo. By injecting IFITM3-overexpressing or control AGS cells into tail vein of severe combined immunodeficiency (SCID) mice, we demonstrated that overexpression of IFITM3 significantly elevated pulmonary metastasis (Fig. 3L).

### Depletion of IFITM3 significantly impedes cancer stemness and chemoresistance of GC cells

**Fig. 4:**
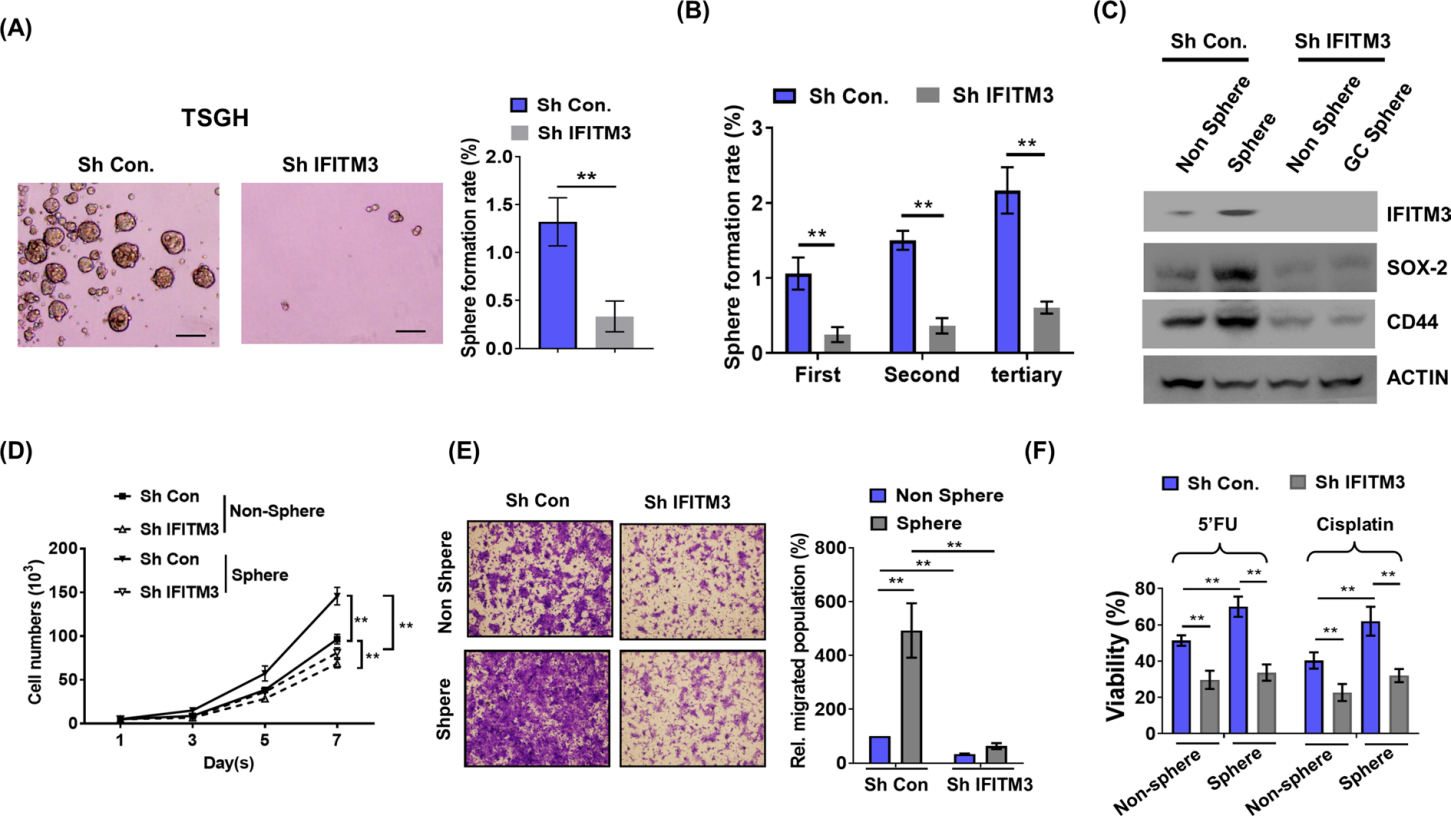
Silencing of IFITM3 diminishes stemness of GC and impairs proliferation and chemoresistance to 5’FU and cisplatin. **A** Knockdown of IFITM3 was conducted in IFITM3 high-expressing TSGH cells to assess its impact on sphere formation rate (Left panel, scale bar, 500 μm; right panel is the statistical results, **p < 0.01). **B** Serial passaging on sphere formation was utilized to determine self-renewal abilities of *IFITM3*-silenced cells of TSGH cells (**p < 0.01). **C** IFITM3, SOX-2 and CD44 protein expressions were determined by western blots in non-sphere cells and spheres formed from TSGH cells with or without IFITM3 knockdown (**p < 0.01). Cell growth curves (**D**) or migration rates (**E**) were determined from measurements on viable or migrated cells from non-spheres or spheres that had been treated with shCon or shIFITM3 (**p < 0.01). **F** Chemoresistance of the indicated groups were determined based on relative number of cells survived after treatment with 5’FU (20 μg/ml) or cisplatin (3 μM) for 72 h (**p < 0.01)

In view of the observation that IFITM3 was involved in chemoresistance, we continued to ascertain whether the function of IFITM3 in mediating cancer stemness contributed to the 5’FU and cisplatin chemoresistance in GC. The role of IFITM3 was further characterized by silencing IFITM3 in TSGH cells that are IFITM3 high-expressing and by examining for changes in sphere forming capability. Our results showed that IFITM3 silencing that resulted in near ablation of IFITM3 protein expression substantially reduced spheres forming capacity (Fig. 4A). To corroborate the effects of IFITM3 on GC-CSC, spheres of IFITM3-silenced and control TSGH cells were serially passaged in order to determine *bona fide* IFITM3-mediated stemness regultation. After three serial passages, sorted IFITM3-silenced cells persistently demonstrated significantly lower self-renewal capacities than sorted control cells (Fig. 4B). To characterize IFITM3-associated GC-CSC regulation, we scrutinized differences between spheres and non-spheres in expression of canonical CSC markers such as SOX-2 and CD44. As shown in Fig. 4C, the expression of CD44 and SOX-2 were both increased in the GC-spheres as compared to that in non-spheres. In contrast, IFITM3 silencing led to downregulation of CD44 and SOX-2 in the GC-spheres but not in the non-spheres (Fig. 4C). These data suggested the capability of IFITM3 in regulating GC-CSC was through modulating expression of SOX-2 and CD44. In addition, silencing IFITM3 in the non-spheres and GC-spheres consistently led to significantly lowered cellular proliferation (Fig. 4D). Similarly, cellular migration of both non-spheres and GC-spheres were largely compromised when IFITM3 expression was absent in the IFITM3-silenced cells as compared to those of sorted control cells (Fig. 4E).

Novel transmembrane proteins such as IGF-1R is known for their role in modulating cancer stemness and resistance to chemotherapy [30]. We thus hypothesized that IFITM3-mediated chemoresistance in GC could be attributed to the GC-CSCs and sphere formation capabilities directly regulated by IFITM3 expression. Sphere-sorted cells that were *IFITM3*-silenced were subsequently subjected to the treatment of 5-FU or cisplatin. Compared with control cells, IFITM3-silenced TSGH cells displayed significantly lower survival rates in both non-sphere and GC-sphere groups that received 5’FU or cisplatin treatment (Fig. 4F).

### IFITM3 associate with MET/AKT complex to regulate HGF triggered GC progression

**Fig. 5:**
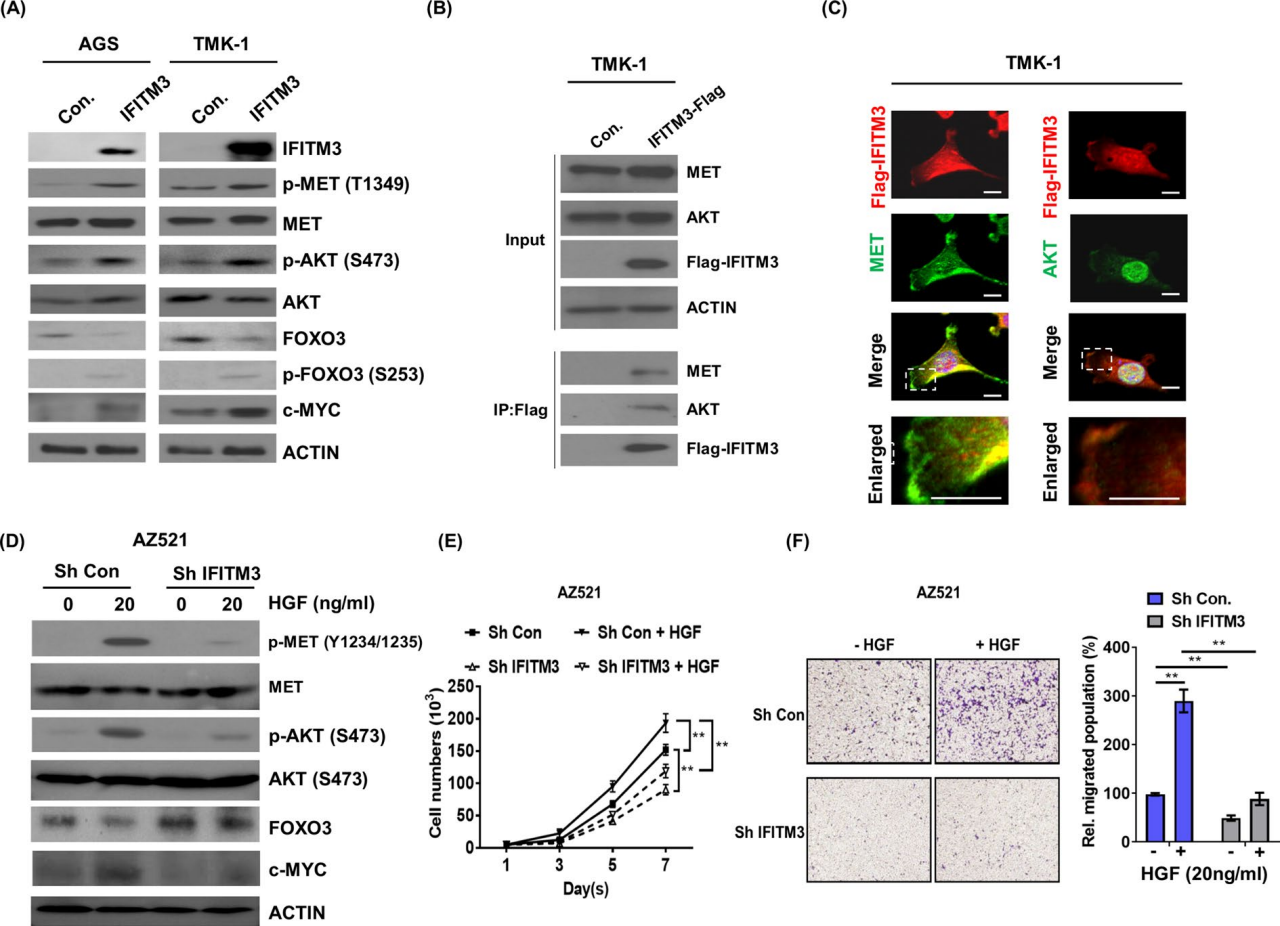
IFITM3 associates with MET/AKT complex in GC to transmit HGF signal and cancer progression. **A** Impacts of IFITM3-overexpression on signaling molecules MET, AKT, FOXO3 and c-MYC were determined by their phosphorylation status on western blots. **B** Protein extracts of Flag-IFITM3-overexpressing or control TMK-1 cells were immunoprecipitated with Flag antibody and analyzed for protein–protein interaction with MET and ATK on western blots. **C** Immunofluorescent imaging was conducted to show cellular co-localization between Flag-IFITM3 and MET or AKT. **D** Control or IFITM3-silenced AZ521cells that had been treated with or without HGF (20 nM) were used to assess the influences of IFITM3 on phosphorylation of MET and AKT, FOXO3 and c-MYC expressions. Cellular growth curves (**E**) and migration rates (**F**) of the indicated groups of AZ521 cells as in (**D**) were determined by measurements of viable or migrated cells after treatments (Right panel is the statistical results, **p < 0.01)

Previous studies reported that IFITM3 served as a PIP3 scaffold engaged to amplify PI3K/AKT signaling in B cells [23], while activation of MET via binding to HGF was found to enhance cancer cell proliferation, metastasis as well as resistance to regimens like chemotherapies through triggering of downstream PI3K/AKT signaling [31–33]. Based on these findings, we next investigated whether IFITM3 could associate with HGF/MET signaling complex to execute its oncogenic function in GC. Firstly, we observed that overexpression of IFITM3 largely augmented the phosphorylation of MET (Y1234/1235) and AKT (S473) in the endogenously IFITM3 low-expressing AGS and TMK-1 cells (Fig. 5A). FOXO3 has been reported as a crucial transcriptional factor involved in tumor suppression [34]. Activation of HGF/MET/ATK signaling pathway can cause the phosphorylation of FOXO3 on Thr32, Ser253, and Ser315, leading to FOXO3 export form the nucleus of cells [8, 35]. After the cytoplasmic retention, FOXO3 is ubiquitinated and subsequently degradated by proteasome[36]. We observed that FOXO3 was greatly decreased and phospho-FOXO3 (S253) was increased in IFITM3-overexpressing GC cells (Fig. 5A). Moreover, a crucial downstream effector of HGF/MET/ATK/FOXO3, c-MYC[37] [38], was also greatly upregulated in IFITM3-overexpressing GC cells (Fig. 5A). These findings in IFITM3-mediated phosphorylation events hence raised an important question if IFITM3 associates with MET/AKT signaling complexes to promote oncogenesis in GC. Interestingly, our co-immunoprecipitation (co-IP) experiments demonstrated that when Flag-tagged IFITM3 was overexpressed in TMK-1 cells, IFITM3-Flag clearly interacted with MET as well as AKT (Fig. 5B), implicating the role of IFITM3 in activating HFG/MET/PI3K/ATK signaling complexes. Further, these results were substantiated by our immunofluorescence (IF) staining analyses that confirmed partial colocalization of IFITM3 with MET as well as AKT signaling complex (Fig. 5C). To further substantiate functional roles that IFITM3 plays in regulating HGF/MET and AKT signaling, HGF treatments were delivered to control or IFITM3-silenced AZ251 GC cells that were IFITM3 high-expressing. The results showed that silencing of IFITM3 significantly suppressed HGF-induced MET and AKT phosphorylation, FOXO3 downregulation and c-MYC upregulation (Fig. 5D). Further functional analyses showed that HGF-stimulated cellular proliferation and migration were both significantly repressed when IFITM3 expression was silenced (Fig. 5E, F). These observations strongly suggested that IFITM3 is critical to HGF-triggered oncogenic signaling in GC via associating with MET/AKT signaling complex.

### IFITM3 promotes oncogenesis of GC via suppression of FOXO3

**Fig. 6:**
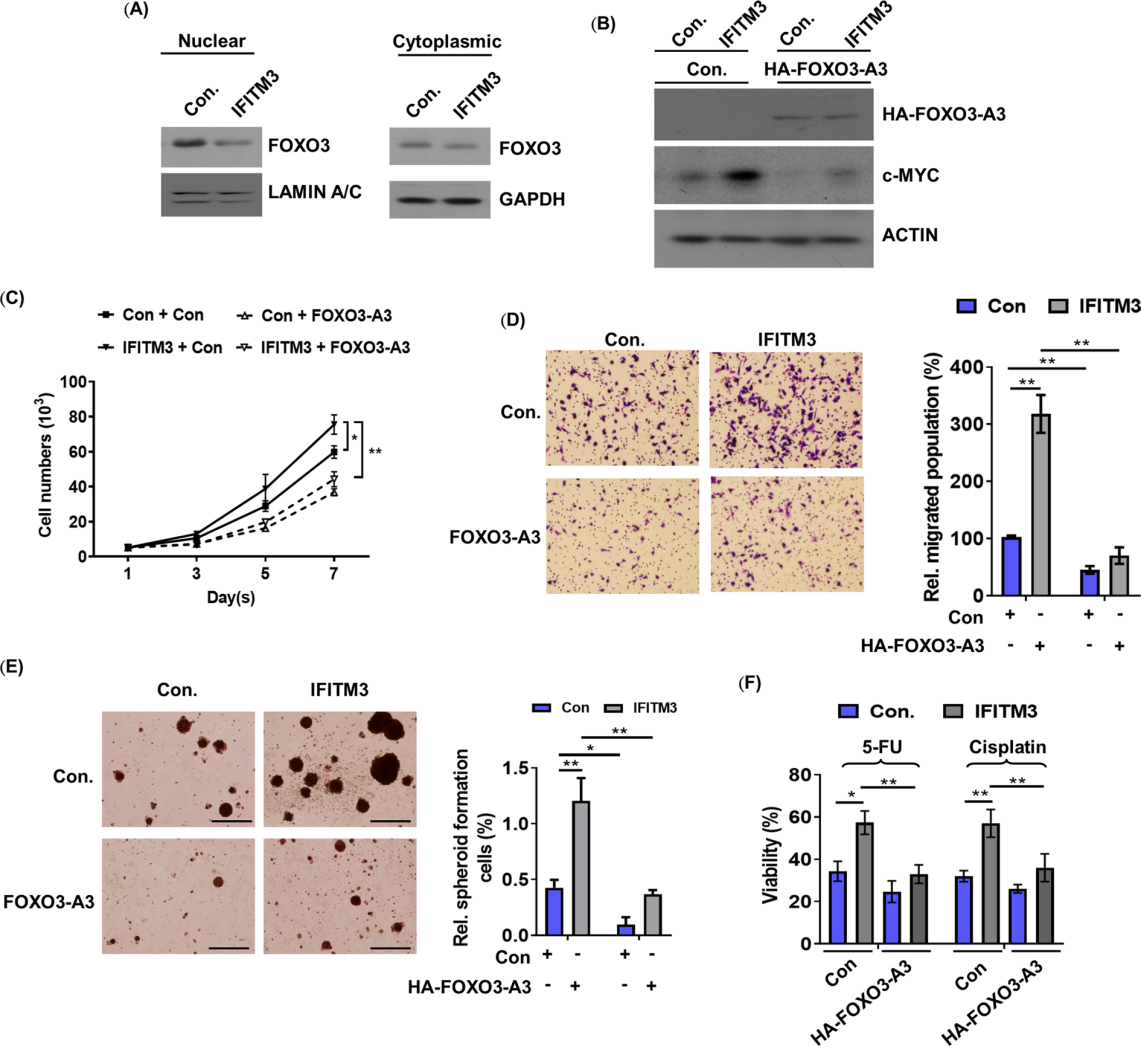
IFITM3 suppress nuclear expression of FOXO3 to promote c-MYC expression and GC progression. **A** Immunoblotting analysis of FOXO3 in the nuclear and cytoplasmic fractions of IFITM3-overexpressing or control TMK-1 cells. **B** Immunoblotting analysis of c-MYC in IFITM3 overexpressing or control TMK-1 cells with HA-FOXO3-A3 overexpression. Cellular growth curves (**C**) and migration rates (**D**) of the indicated groups of TMK-1 cells as in (**B**) were determined by measurements of viable or migrated cells after treatments (*p < 0.05, **p < 0.01). **E** Sphere formation assays were performed to assess the influences of HA-FOXO3-A3 overexpression on IFITM3-overexpressing TMK-1 cells (Left panel, scale bar, 500 μm; right panel is the statistical results. *p < 0.05, **p < 0.01). **F** Chemosensitivities of the same indicated groups of TMK-1 cells were determined based on the relative number of cells survived after treatment with 5’FU (20 μg/ml) or cisplatin (3 μM) for 72 h. *p < 0.05, **p < 0.01

To determine whether IFITM3 mediated c-MYC upregulation is required for FOXO3 suppression, FOXO3 expression was evaluated in IFITM3-overexpressing TMK cells. Overexpression of IFITM3 resulted in downregulation of FOXO3 in the nuclear extracts (Fig. 6A). In addition, overexpression of HA-tagged FOXO3-A3, a constitutively active form of FOXO3 (in which the three AKT phosphorylation sites were altered to alanine), significantly blocked IFITM3 induced c-MYC expression in the cells (Fig. 6B), suggesting that IFITM3 could suppress nuclear FOXO3 expression to induce c-MYC expression.

To further clarify the influence of FOXO3 on IFITM3-mediated GC progression and therapeutic resistance, the characteristics of CSCs in IFITM3-overexpressing GC cells following FOXO3-A3 overexpression were determined. Overexpression of FOXO3-A3 efficiently blocked IFITM3 and triggered GC proliferative and metastatic capacities as well as sphere forming ability (Fig. 6C–E). Our results further demonstrated FOXO3-A3 overexpression efficiently suppressed IFITM3 enhanced chemoresistance (Fig. 6F), collectively implying that the IFITM3-mediated oncogenic effects were dependent on FOXO3 suppression.

### IFITM3 promotes oncogenesis of GC via MET/AKT/c-MYC signaling axis

**Fig. 7:**
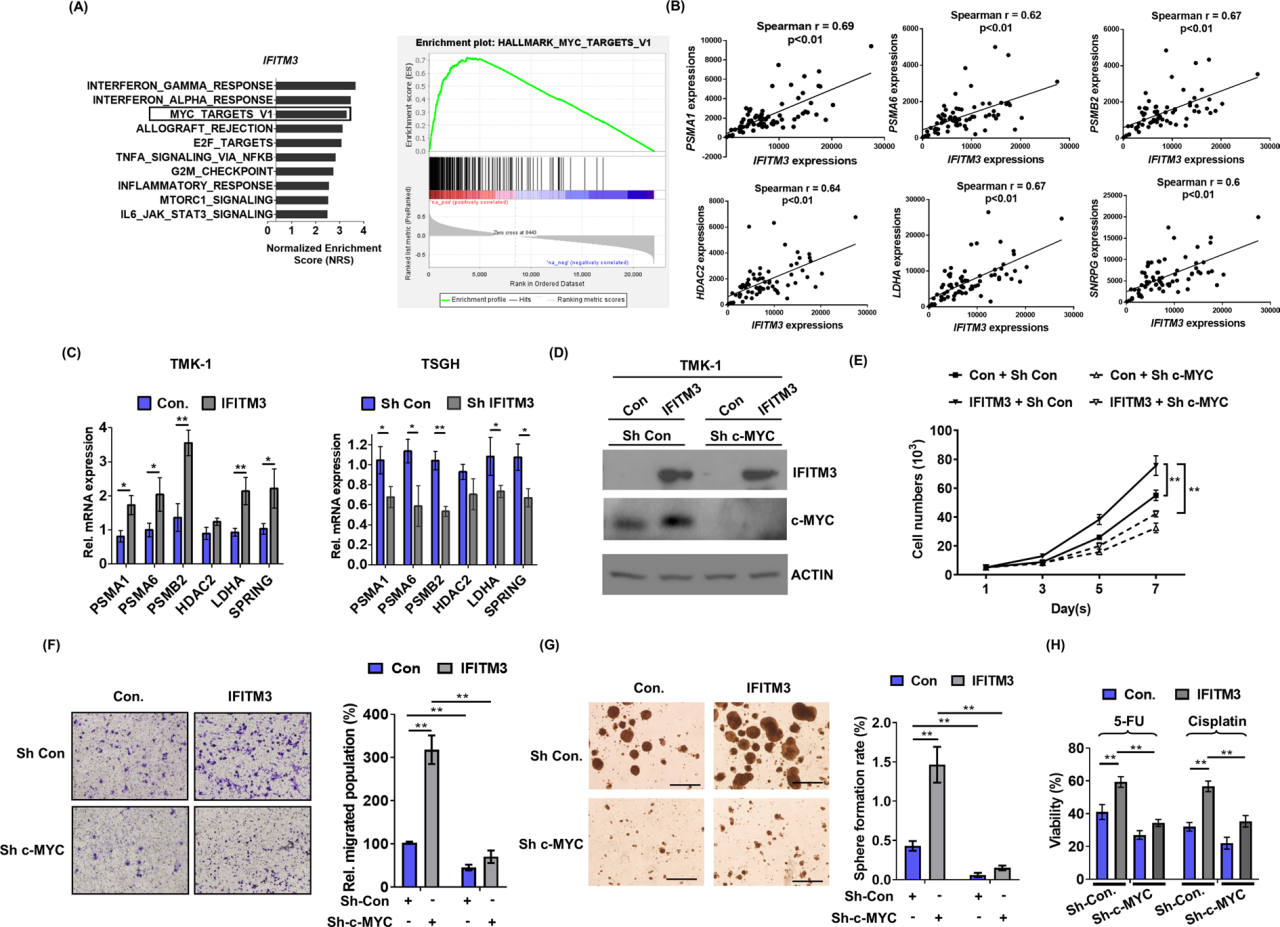
c-MYC and its target genes are regulated by IFITM3 in GC oncogenesis and chemoresistance. **A** Gene Set Enrichment Analysis (GSEA) was carried out to analyze the co-expression network and determine Pearson’s correlation coefficient between IFITM3 and other hallmarks of cancer in cBioPortal. The right panel represents an enrichment plot of IFITM3-correlated genes in c-MYC-regulated targets. **B** Spearman rank correlation coefficient of individual genes (including *PSMA1*, *PSMA6, PSMB2*, *HDAC2*, *LDHA* and *SPRING*) against *IFITM3* in 71 GC tissues from Cho Gastric dataset (GSE138861). **C** Quantitative RT-PCR analyses were conducted on the six c-MYC target genes for their gene expression in TMK-1 IFITM3-overexpression or TSGH IFITM3-depletion models (*p < 0.05, **p < 0.01). **D** Western blot analyses of IFITM3 and c-MYC expression in control or IFITM3-overexpressing TMK-1 cells with or without c-MYC knockdown. Cellular growth curves (**E**) and migration rates (**F**) of the same experimental groups were determined by measuring viable and migrated cell numbers, respectively. **G** Sphere formation assays were performed to assess the influences c-MYC knockdown on IFITM3-overexpressing TMK-1 cells. (Left panel, scale bar, 500 μm; right panel is the statistical results, **p < 0.01) **H** Chemosensitivities of the same indicated groups of TMK-1 cells were determined based on the relative number of cells survived after treatment with 5’FU (20 μg/ml) or cisplatin (3 μM) for 72 h. *p < 0.05, **p < 0.01.

**Fig. 8:**
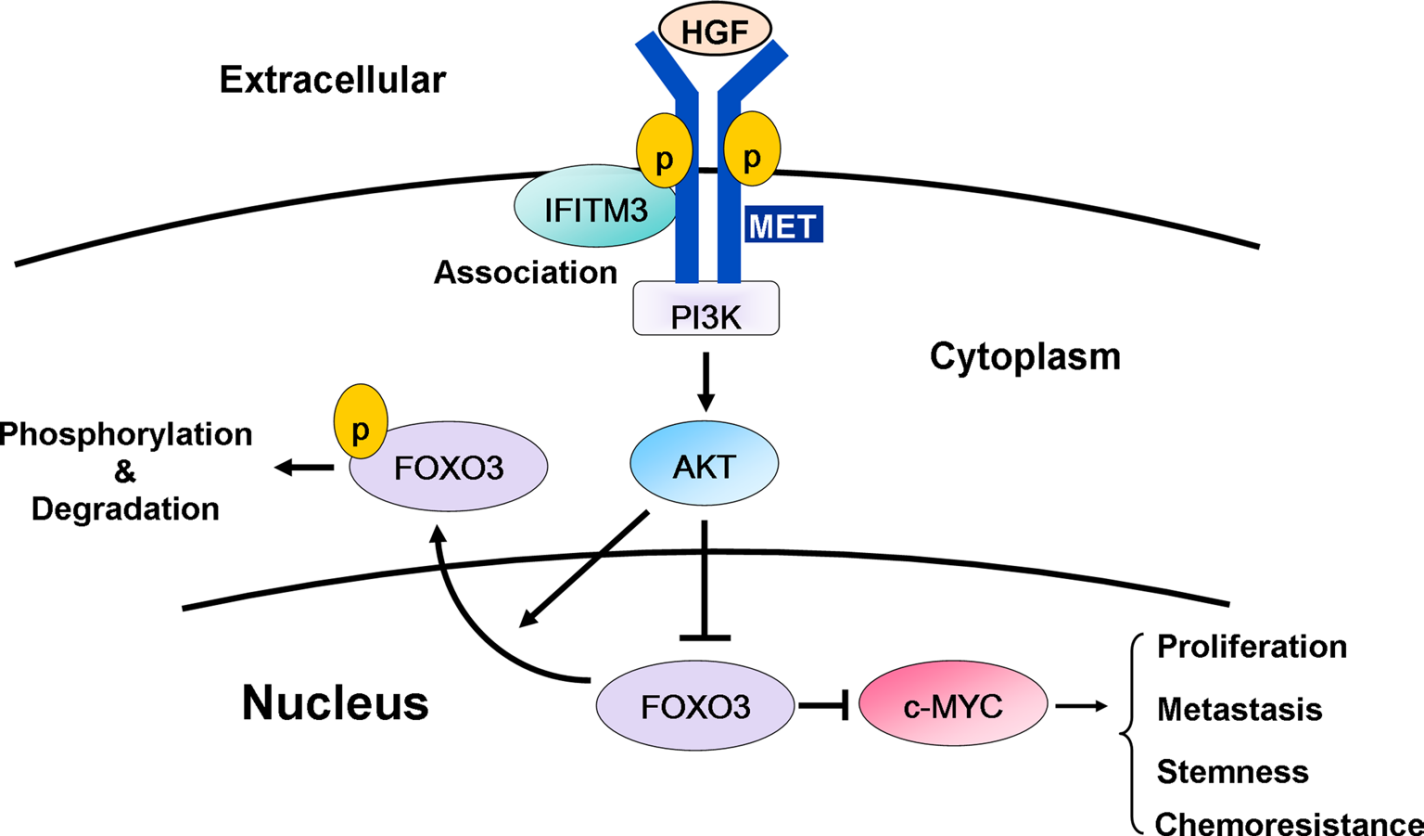
Cartoon illustration that depicts a model of IFITM3-mediated MET/ AKT/FOXO3/c-MYC cascade signaling pathway. Data presented in this study reveals a new signaling model in which IFITM3 associates with MET and AKT complex, leading to enhanced HGF/MET signaling as well as suppression of FOXO3, consequently resulting in upregulation of c-MYC to promote proliferation, metastasis, cancer stemness and chemoresistance of GC cells

To further delineate MET/AKT signaling events mediated IFITM3 and identify downstream effectors, we determined the Pearson’s correlation coefficient between IFITM3 and other genes in cBioPortal (TCGA Provisional) using Gene Set Enrichment Analysis (GSEA). The bioinformatical analyses revealed the co-expression network of IFITM3 and hallmarks of target genes associated with IFITM3 based on normalized enrichment scores. As shown in Fig. 7A, the signature genes positively correlated with IFITM3 are characteristic of tumor-promoting functions, including MYC targets. Further, we analyzed the 415 overexpressed proteins in GC tumors from our proteomic database with MetaCore software, and our results revealed that signature genes involved in cell cycle regulation, metabolism and transcriptional regulation (Additional file 1: Fig. S1A). Interestingly, MCM2 to MCM7 (Minichromosome Maintenance) proteins, the critical proteins regulated by c-MYC during cell division [39–41], were identified to be significantly upregulated in GC tissues as compared to adjacent normal gastric tissues (Additional file 1: Fig. S1B). In addition to MCM2 to MCM7, six downstream oncogenic targets of c-MYC implicated in cancer survival and metabolism including *PSMA1*, *PSMA6, PSMB2*, *HDAC2*, *LDHA* and *SPRING* [42–45] were positively correlated with *IFITM3* in 71 GC specimens using the Cho dataset [27] (Fig. 7B, and Additional file 1: Fig. S1C). Consistently, the gene expressions of *MCM2*, *MEM3*, *MCM4*, *PSMA1*, *PSMA6, PSMB2*, *LDHA* and *SPRING* were significantly elevated in *IFITM3*-overexpressing TMK-1 cells, while reduced expressions were observed in *IFITM3*-silenced TSGH cells (Fig. 7C and Additional file 1: Fig. S1D), further supporting IFITM3 as a positive regulator in MET/AKT/c-MYC signal axis in GC. To elaborate c-MYC induction is required during IFITM3-mediated tumor progression and chemoresistance acquisition, a *c-MYC*-silenced model was established using IFITM3-overexpressing GC cells (Fig. 7D). Our data demonstrated that both cellular proliferation and migration abilities of IFITM3 high-expressing cells were significantly compromised by the knockdown of c-MYC (Figs. 6F, 7E). Further, the diminished sphere formation by knockdown of c-MYC in IFITM3-overexpressing cells suggested c-MYC as a crucial target of IFITM3 (Fig. 7G). In line with these observations, c-MYC knockdown significantly lowered the 5’FU and cisplatin chemoresistance attributed by overexpression of IFITM3, confirming the crucial function of c-MYC in mediating the oncogenic effects of IFITM3 (Fig. 7H). Collectively, our present study revealed a novel mechanism modulated by IFITM3-associated HGF/MET/AKT signaling complexes that suppressed FOXO3, consequently leading to c-MYC upregulation to promote cell proliferation, metastasis, stemness and chemoresistance in GC (Fig. 8).

## Discussion

Consistent with literature reports that showed IFITM3 as a prognostic marker associated with progression of head and neck squamous cell carcinoma [46] and acute myeloid leukemia [47], our study also illustrated that overexpression of IFITM3 was closely correlated with advanced pathological stages in GC. Despite chemoresistance and CSCs have been known for their collective detrimental influences that often lead to low 5-years OS rate in advanced GC patients [2, 48], underlying molecular mechanisms that can be strategized for effective therapeutics still remain largely veiled. There is thus an urgent need to discover novel mechanisms and targets to conquer cancer stemness and chemoresistance for GC patients. To date, there is only one report that suggested IFITM3 as one of the significantly upregulated CSCs-related genes in MDA-MB-231 breast cancer cells by single-cell RNA sequencing [49]. In present study, we unraveled IFITM3 for the first time as a critical mediator that promotes chemoresistance by elevating cancer stemness of GC. This is evident from ectopic expression of IFITM3 that significantly increased sphere forming ability, whereas IFITM3 knockdown largely reduced the sphere forming ability and CSC markers in GC lines (Figs. 3D, H, 4A–C). This finding of IFITM3-mediated cancer stemness was demonstrated to contribute to 5’FU- and cisplatin-associated chemoresistance in four GC cell lines of varying IFITM3 expression (Figs. 2I–J, 3I–J, 4F, 6H). These results provided the first evidence that shows IFITM3 acts not only as a cancer stemness regulator but also contributes to the development of chemoresistance in GC.

Although IFITM3 had been reported to engage in different regulatory signaling pathways to exert oncogenic functions in several other cancer types, IFITM3 was only suggested to be a potential target of the Wnt/β-catenin signaling in gastric cancer [50]. In prostate cancer, for instance, IFITM3 facilitated tumor progression and bone metastasis via TGF-β-Smads-MAPK pathway [18]; as IFITM3 was proposed to serve as a PIP3 scaffold protein to amplify PI3K signaling in B cells [23]. In present study, our data demonstrated that IFITM3 can not only associate with HGF/MET but also AKT signaling complexes to execute its oncogenic functions via c-MYC and its target genes (Figs. 5 and 6). The only report that showed the ability of IFITM3 in regulating c-MYC expression was via ERK1/2 signaling to promote tumorigenesis in HCC [21], in which IFITM3 was also reported to enhance the invasiveness and metastasis of HCC through regulation of p38/MAPK/MMP9 signaling [17].

## Conclusions

c-MYC is a transcription factor reported to constitutively and aberrantly expressed in more than 70% of human cancers, and has attracted enormous public efforts in identifying new small molecule inhibitors for therapeutic use in clinics. Oncogenesis driven by the signaling of HGF-induced MET/PI3K/AKT/c-MYC has been well-characterized in many different cancer types [31, 51]. Nonetheless, tens of HGF/MET-based or c-MYC-targeting drugs that entered clinical trial have recently returned with unsatisfactory results [52, 53]. In contrast, our present data unprecedentedly reported that IFITM3 can associate with HGF/MET/AKT to promote phosphorylation and nuclear exclusion of FOXO3. After the cytoplasmic retention, FOXO3 is ubiquitinated and subsequently degradated by proteasome[36]. It may consequently result in induction of c-MYC targets to promote GC proliferation, migration, stemness, metastasis and drug resistance. Over the past two decades, in fact, the notion of dual-targeting has become increasingly popular since many therapeutics that reply on solely one signaling pathway or drug target often led to disease relapse, drug resistance or eventually metastasis [54, 55]. In addition to dual-targeting common signaling pathways such as PI3K/mTOR or p53/MDM2/MDMX [56, 57], potent and selective inhibitors that target MET and other signaling molecule such as SMO, ALK (Anaplastic lymphoma kinase) have also recently been reported [53, 58, 59]. Therefore, the unique characteristic of IFITM3 in mediating crosstalk between HGF-induced MET and c-MYC signaling pathways indeed warrants future IFITM3-based drug designs.

## Methods

### Subjects

All patients diagnosed pathologically with gastric cancer (GC) at the Chang Gung Memorial Hospital (CGMH) from 2000 to 2005 were enlisted after informed consent. None of the patients had received radiotherapy and chemotherapy before surgery and all were undergone gastric resection. All the pathologic analyses and biological examination were conducted with informed consent. The research protocol was approved and verified by the Medical Ethics and Human Clinical Trial Committee of the Chang Gung Memorial Hospital (IRB NO. 201702000B0C101). The patients were followed regularly at the outpatient department in the Chang Gung Memorial Hospital every 3 months in the first 2 years, every 6 months between 3 to 5 years, and once a year thereafter.

### Cell lines and cultures

The human GC cell line, AGS and AZ521 were provided by Professor Yu-Sun from Chang Gung University, Taoyuan, Taiwan). The human GC cell line, TMK-1, was provided by Professor Tsu-Chung Chang from National Defense Medical Center, Taipei, Taiwan. All GC cell lines were cultured in RMPI 1640 (Invitrogen) supplemented with 10% FBS at 37˚C in a humidified atmosphere containing 5% CO_2_. These cell lines were authenticated using short tandem repeat-based assay with the Promega StemElite ID System. Cells were maintained in culture for no longer than 3 months and routinely checked for mycoplasma contamination.

### Immunoblot analysis

Western blot was performed as described previously [60]. The following primary antibodies were used at the recommended dilutions: anti-MET (Santa Cruz Biotechnology, SC-8057), anti-phospho-MET (Cell signaling technology, 3077S), anti-AKT (Santa Cruz Biotechnology, SC-5298), anti-phospho-AKT (Abcam, ab81283), anti-c-MYC (Abcam, ab32072), anti-Flag (Sigma-Aldrich, F3165), anti-HA (Santa Cruz Biotechnology, sc-7392), anti-FOXO3 (Santa Cruz Biotechnology, SC-48348), ABclonal phospho-FOXO3 Rabbit mAb ABclonal Inc., USA, ABclonal IFITM3 Rabbit pAb ABclonal Inc., USA, anti-CD44 (GeneTex, GTX102111), anti-SOX2 (GeneTex, GTX101506) anti-ACTIN (Chemicon, MAB1501). All the experiments were reproducible and repeated at least three times.

### Cell proliferation assay

The cells cultured in RPMI containing 10% FBS were used for seeding at a density of 5 × 10^3^ cells/well and the cell proliferation rates determined by staining with 0.4% Trypan blue solution using the LUNA Automated Cell Counter at the indicated times. For colony formation, GC cells (5 × 10^3^ cells/well) were seeded and cultured for 10 days. Subsequently, the colonies were stained with crystal violet. Experiments were performed in triplicates, and the results are presented as means ± SEM.

### Transwell migration assay

For the transwell in vitro assay, 1 × 10^5^ GC cells in 100 μL RPMI medium were added to each upper chamber, whereas the lower chamber contained serum free medium supplemented with 20% (v/v) FBS. Following incubation for 16 h at 37 °C, cells traversing the filter to attain the lower chamber were stained and counted. All assays were repeated at least three times. Among-treatment differences were evaluated using Student’s t-test (*p < 0.05; **p < 0.01). Experiments were performed at least three times, and the results are presented as means ± SEM.

### Immunofluorescence and Immunohistochemistry staining

The procedure of fixation and antibodies staining for immunofluorescence and immunohistochemistry were performed as described [61]. For the immunofluorescence analysis, the images were acquired using Zeiss Apotome fluorescence microscope and AxioVision Rel. 4.7 software. (Carl Zeiss, Gottingen, Germany). The following primary antibodies were used at the recommended dilutions: anti-Flag (Sigma-Aldrich, F3165), anti-MET (Abcam, ab51067) and anti-AKT (Cell signaling technology, SC-5298). Images were acquired using a Carl Zeiss Axiovert 200 M microscope and AxioVision Rel. 4.7 software. For immunohistochemistry staining, ABclonal IFITM3 Rabbit pAb ABclonal Inc., USA, was used at the recommended dilutions. The avidin–biotin complex method and scoring formula were employed according to the previously described method [62]. The IHC staining intensities were graded as absent (0), weak (1 +), medium (2 +) and strong (3 +). The histoscore (Q) was calculated with multiplying the percentage (P) of positive GC cells by the intensity (I) according to the formula: Q = P1 x I1 + P2 x I2 + Pn x In.

### RNA interference

Lentiviral pLKO, scramble and IFITM3 shRNAs were obtained from the National RNAi Core Facility at the Institute of Molecular Biology/Genomic Research Center, Academia Sinica. The recombinant lentiviruses carrying shRNAs were produced according to the previously described method [62] by transfecting 293FT cells with the plasmid mixture consisting of pLKO-shRNA vectors, pCMV-ψR8.91 (Gag/Pol/Rev) and pMD-G (VSV-G envelope), using Lipofectamine 2000 (Invitrogen) in keeping with the manufacturer’s recommendations. These plasmids were extracted using the DNA extraction kit (Biotools Co., Ltd., Taipei, Taiwan). The virus-containing culture mediums were collected three days following transfection and used to infect GC cells in combination with polybrene (10 µg/ml) (Sigma-Aldrich, St. Louis, MO, USA). Western blot was used to determine the effects of gene silence.

### Coimmunoprecipitation

Five mg of indicated cell extract was incubated with 2 mM dithiobis[succinimidylpropionate] (DSP) (22,586, Thermo Fisher) in PBS for 10 min and quenched by adding 1 M Tris (pH 7.5) and subsequently terminated by the addition of quenching buffer (20 mM Tris, (pH 7.5) and 100 mM KCl) for 15 min at room temperature. These cell lysates were then precleared using 2 μg of either mouse or goat IgG and Protein *A/G Magnetic Beads* (#15,752,442, Thermo Fisher Scientific) for 1 h at 4 °C on a rotator. The precleared lysates were then subjected to immunoprecipitation by incubating with indicated antibody at 4 °C for 3 h. Next, protein A/G beads were then added and mixed further by incubation on a rotator at 4 °C for 16 h. The protein complexes were further analyzed by immunoblotting with indicated antibodies.

### Tumor sphere formation assay

GC cells were digested with 0.25% trypsin, washed twice with PBS and suspended in the sphere formation medium (+ 100 mg/ml EGF + 100 mg/ml bFGF + 1 ml B27 supplement /50 ml DMEM-F12) and seeded on an ultra-low attachment plate (2000 cells/ml), subsequently cultivated for 7 days, and the spheres counted under a microscope. The size of Oncospheres larger than 100 μm were isolated and counted. The experiments were repeated at least three times, and the results are presented as means ± SEM.

### Xenograft tumor formation

For the tumor propagation assay, IFITM3-overexpressing GC cells were subcutaneously injected into 6–8-week-old male BALB/c nude mice randomly. The tumor volumes were measured twice per week. These groups of mice were sacrificed 28 days after implantation. The tumor formation was assessed and calculated. The sample sizes were based on literatures. All of the experiments were conducted in the observer-blinded and randomized manner. All procedures were performed in accordance with the Guide for Care and Use of Laboratory Animals issued by the Institutional Animal Care and Use Committee of Chang Gung University and the National Institutes of Health of United States (CGU106-142).

### Real-time RT-PCR

To quantify IFITM3 transcripts in GC, total RNAs were extracted from GC cells and clinical tissues with RNeasy mini Kit (QIAGEN Inc., Dusseldorf, Germany). Then, these RNAs were converted into cDNA using MMLV reverse transcriptase (Thermo Fisher Scientific Inc., Kalamazoo, USA) after DNase (QIAGEN Inc., Dusseldorf, Germany) pre-treatment. Q-RT-PCR was performed using the SYBR Green system (Biotools Co., Ltd., Taipei, Taiwan). Fluorescence emitted by SYBR Green was observed using the ABI PRISM 7500 sequence detection system (Applied Biosystems, Werrington, UK). To further eliminate the interference of genomic DNA in qRT-PCR analysis, we designed the PCR primers that span exon-exon junctions of specific genes for detecting the expressions of *IFITM3* in clinical GC specimens and *MCM2*, *MCM3*, *MCM4*, *MCM5*, *MCM6*, *MCM7*, *PSMA1*, *PSMA6*, *PSMB2*, *HDAC2*, *LDHA* and *SPRING* in IFITM3-overexpressing or silenced GC cells. 18S ribosomal RNA is used as control for qRT-PCR analyses. The Q-RT-PCR experiments in GC cell lines were performed in triplicates, and the results are presented as means ± SEM. The primer information is listed below: IFITM3: (F5’-GGCTTCATAGCATTCGCCTACT-3’, R5’-TCACGTCGCCAACCATCTT-3’), MCM2: F5'-GGCGGAGAGGATCGTGGTA, R5'-TGGATGCCATGGTGAAGGAT, MCM3: F5'-GCGGGAGGCTCAGAGAGAT, R5'-TCTGATAAATTCCCTGGTCTTCCT MCM4: F5'-CCCTCCCCAAATGCATTCT, R5'-CGTATGTCAGTGGTGAACTAACATCA, MCM5: F5'-AAGGAGTTCCTGCGGCAGTA, R5'-CGCTTGAGTTCATCCCTGTATTT,MCM6: F5'-GTTCCTGGACTTCTTGGAGGAGTT, R5'-AATCAGTTCCTCTGCTAATTGCAA, MCM7: F5'-GGTGGTGGCCACTTACACTTGT, R5'-GCACATGATCAGAGGCATGAAA, PSMA1 (F5’-ATGGGCCCTCACATTTTCC-3’, R5’-CATGGCTCTGCAGTCAAAATAGTT-3’), PSMA6: (F5’-CGAGGGTCGGCTCTACCAA-3’, R5’-TTTCCCTCTGACAGCTACTGATGT-3’, PSMB2: (F5’-TGTCCAGATGAAGGACGATCAT-3’, R5’-CCTCTCCAACACACAGGAGTAAT-3’), HDAC2 (F5’-CAAGGAGGCGGCAAAAAA-3’, R5’-GGGATGACCCTGTCCATAATAATAA-3’, LDHA (F: 5’-CCGCCCGACGTGCAT-3’, R: 5’-TCCTTTAGAGTTGCCATATTGGACTT-3’, SPRING (F: 5’-GTACAGCTGCGCAACATGGT-3’, R: 5’-AGACAGGCTTCAAACTCCACACT-3’), and 18S rRNA (F: 5’-GAGCCGCCTGGATACC-3’, R: 5’-CCTCAGTTCCGAAAACCAACAA-3’).

## Statistical analysis

All the in vitro experiments were reproducible and repeated at least three times, and the results are presented as means ± SEM. The Statistical analysis was performed with the GraphPad Prism software (GraphPad Software, CA) using Mann–Whitney U or Fisher’s exact test for between-group comparisons. The two-tailed paired or unpaired t-tests were performed to determine the significance between the groups compared. P values < 0.05 were considered statistically significant.

## Supplementary Information

**Additional file 1: Fig. S1.** MCM2-7 proteins are regulated by IFITM3 in GC proliferation. (A) MetaCore analysis of 415 overexpressed proteins in GC tumors from our proteomic database. The signature genes involved in cell cycle regulation, metabolism and transcriptional regulation were shown. (B) Relative MCM2-7 proteins in GC tissues as compared to adjacent normal gastric tissues from our iTRAQ quantitative proteomic analysis (C) Spearman rank correlation coefficient of individual genes (including MCM2, MCM3, MCM4, MCM5, MCM6 and MCM7) against IFITM3 in 71 GC tissues from Cho Gastric dataset (GSE138861). (D) Quantitative RT-PCR analyses were conducted on MCM2-7 genes for their transcript expression in TMK-1 IFITM3-overexpression or TSGH IFITM3-depletion models (*p<0.05, **p<0.01).

**Additional file 2: Table S1.** The 415 overexpressed proteins in GC tumors from our iTRAQ quantitative proteomic database.

**Additional file 3: Table S2.** The repeated presented protein candidates in iTRAQ quantitative proteomic analysis.

## Data Availability

The experimental procedure and results of iTRAQ quantitative proteomic analysis have been organized and deposited to Figshare (10. 6084/m9.figshare.20069969). Data could be available upon request to interested researchers. Please send data requests to Hsiang-Cheng Chi, PhD. Graduate Institute of Integrated Medicine, China Medical University, Taichung, Taiwan.

## Abbreviations

GC: Gastric cancer
OS: Overall survival
MET: Mesenchymal-epithelial transition factor
HGF: Hepatocyte growth factor
CSCs: Cancer stem cells
IFITM3: Interferon induced transmembrane protein 3
shRNA: Hairpin RNA
HCC: Hepatocellular carcinoma
FOXO3: Forkhead Box O3
c-MYC: MYC proto-oncogene
ALK: Anaplastic lymphoma kinase
