# Supplementary material for: IFITM3 promotes malignant progression, cancer stemness and chemoresistance of gastric cancer by targeting MET/AKT/FOXO3/c-MYC axis
Source: Cell Biosci. 2022 Aug 8;12:124. doi: 10.1186/s13578-022-00858-8 (PMC9361616; doi:10.1186/s13578-022-00858-8)
Supplement: Supplementary file 1 — Additional file 1: Fig. S1. MCM2-7 proteins are regulated by IFITM3 in GC proliferation. (A) MetaCore analysis of 415 overexpressed proteins in GC tumors from our proteomic database. The signature genes involved in cell cycle regulation, metabolism and transcriptional regulation were shown. (B) Relative MCM2-7 proteins in GC tissues as compared to adjacent normal gastric tissues from our iTRAQ quantitative proteomic analysis (C) Spearman rank correlation coefficient of individual genes (including MCM2, MCM3, MCM4, MCM5, MCM6 and MCM7) against IFITM3 in 71 GC tissues from Cho Gastric dataset (GSE138861). (D) Quantitative RT-PCR analyses were conducted on MCM2-7 genes for their transcript expression in TMK-1 IFITM3-overexpression or TSGH IFITM3-depletion models (*p<0.05, **p<0.01). [file 13578_2022_858_MOESM1_ESM.docx]

**Additional material**

**IFITM3 promotes malignant progression, cancer stemness and chemoresistance of gastric cancer by targeting MET/AKT/FOXO3/c-MYC axis**

**Running title:** IFITM3/MET/c-MYC axis promotes GC progression
Pei-Yi Chu*^1,2,3,4,5^, Wei-Chieh Huang*^6,7^, Shiao-Lin Tung^8,9^, Chung-Ying Tsai^10^, Chih Jung Chen^11,12,13^, Yu-Chin Liu^14,15^, Chia-Wen Lee^16^, Yang-Hsiang Lin^17^, Hung-Yu Lin^18^, Cheng-Yi, Chen^19^, Chau-Ting Yeh^17^, Kwang-Huei Lin*^17,20,21,22^ and Hsiang-Cheng Chi*^6,7^

**Materials and methods of iTRAQ quantitative proteomic anslysis:**

**Clinical Specimens**

Six samples of tumor tissue and adjacent noncancerous mucosa were harvested immediately following gastric resection. Samples were dissected from resected specimens by a pathologist at CGMH. The surgically resected specimens of tumor and their adjacent non-tumor specimens were immediately embedded in O.C.T compound (Tissue-Tek® O.C.T., Sakura Finetek) and stored at -70°C until use. Before LCM experiment, the tissue sections were stained with hematoxylin/eosin staining method and evaluated by pathologists. All subjects signed an Institutional Review Board-approved informed consent prior to study participation.

**LCM and protein extraction.**

Prior to LCM, sixteen-micrometer cyrosections were cut onto membrane slides. The specimens were then fixed in 70% ethanol for 30 sec, washed in 25 % ethanol for 45 sec, placed in Mayer’s hematoxylin solution for 30 sec, rinsed in 75% ethanol, dehydrated in 95% ethanol and twice in 100% xylene for 30 sec, and thoroughly air dried. LCM was performed on Veritas Laser Capture Microdissection and Laser Cutting systems (Arcturus, Mountain View, CA, USA). Briefly, the surroundings of the selected areas were firstly cut by UV laser and then the inside areas were hit by the soft IR laser pulses to dissociate the cut sections from membrane slides. Several selected areas were then adhered on a CapSure LCM Cap (Arcturus) and immediately transferred to a 0.5 ml-microcentrifuge tube for protein extraction. All extracted proteins were dissolved in 50-100 μl lysis buffer (RapiGestTM, Waters) by vortexing at room temperature for 30 min, brief sonication in ice bath, and then centrifugated at 20,000 xg for 10 min to remove insoluble debris. The concentrations of the protein extracts were measured by a modified Bradford assay (Bio-Rad Laboratories, Glattbrugg, Switzerland) and examined by SDS/PAGE and silver staining. The extracts could be stored at -80∘C before next time used.

In solution digestion and iTRAQ labelinllowing basic reverse phase HPLC Total LCM proteins in RapiGest_HEPES buffer (0.1% RapiGest, 20mM HEPES, pH 8.0 ) were reduced with 5 mM tris-(2-carboxyethyl)phosphine (TCEP) at 60 ℃ for 1 h, alkylated with 10 mM methane thiosulfonate at room temperature for 10 min. and subsequently digested with sequencing grade, modified trypsin (Promega, Madison, WI) at an enzyme to substrate ratio 1:100 (W:W) at 37℃ overnight. The enzyme digestion was stopped by adding 0.1% formic acid and further desalted by RP18-microtip as needed for further iTRAQ labeling. Subsequently, peptide extracts were labeled with iTRAQ^TM^ reagents (Applied Biosystems) by adding 10-μl reagent in ethanol and incubation for 1h at 20 ℃ in 60% ethanol, 40 mM triethylammoniumbicarbonate, pH 8.53. After quenching of the reaction with glycine, all labeled extract were combined, mixed and onto a 2x100 mm Germini C18 column (Phenomenex, Torrance, CA) coupled to a Waters HPLC system (Waters Breeze) consisting of a binary solvent delivery pump (model 1525 μ), a dual λ absorbance detector (model 2487). The peptide were eluted using a segmented gradient over 60 min from 5% to 65% solvent B (100% acetonitrile with ammonium, pH 10.0) at a split flow rate of about 0.15 ml/min across the capillary column, followed by isocratic elution at 80% buffer B for 5 min. Solvent A was water at pH 10.0 by ammonia. The effluents were collected to 1 min/tube and pooled into 30~40 fractions dependent on the signal intensity at OD280. Each fraction was dried at vaccum and resuspended in 0.3% formic acid for the following LC-MS/MS analysis.

**Shotgun proteomic identifications**

NanoLC-nanoESi-MS/MS analysis was performed on a Surveyor nanoflow system (Thermo Fisher, San Jose) connected to an LTQ-Orbitrap hybrid mass spectrometer (Thermo Fisher, San Jose, CA) equipped with a PicoView nanospray interface (New Objective, Woburn, MA). Peptide mixtures were loaded onto a 75μm x 100 mm PicoFrit BioBasic C18 capillary column (5μm particle size, 300-Å pore size, New Objective, Woburn, MA) and were separated using a gradient elution over 90 min from 5% to 65% solvent B (100 % acetonitrile with 0.1% formic acid) for at a flow rate of 200 nl/min, followed by isocratic elution at 80% Buffer B for 5 min with a flow rate of 0.25 μl/min across the capillary column. Solvent A was 0.1% formic acid in water.

The Orbitrap mass spectrometer was operated using the XCalibur Developers kit 2.0 in the data-dependent mode. Internal calibration was performed using the ion signal of (Si(CH3)2O)6H+ at m/z 445.120025 as a lock mass. Briefly, survey full scan MS spectra were acquired in the Oribitrap (m/z 350-1600) with the resolution set to 30,000 at m/z 400 and automatic gain control (AGC) target at 106. Maximal ion accumulation time allowed on the LTQ Orbitrap was 1 s for all scan modes; automatic gain control (AGC, target at 106) was used to prevent over-filling of the ion trap. Up to four most intense ion with minimal signal intensity of 20000 were sequentially isolated for MS/MS fragmentation in the order of intensity of precursor peaks and detection in the linear ion trap with previously selected ion dynamically excluded for 120s. The MS/MS spectra were collected by Pulsed Q dissociation (PQD) method under the control of Xcalibur 2.0 software with following settings: normalized collision energy at 30 %, Q activation at 0.7, the activation time of 0.1 and the isolation width at 2.0. The maximum fill time for each MS and MS/MS scan was 100 μs and 100μs, respectively. Four MS/MS microscans were averaged to create proper signal intensity for peptide quantification using iTRAQ reporter ion. Ions with singly charge state were excluded. All the measurements in the Orbitrap were performed with the lock mass option for internal calibration.

The MS and MS/MS raw data were converted to MGF peak list by DTASuperCharge 1.37 (2009-02-19) using SmartPicking with 200 segment size (Th) and max search level of 8. The generated MGF files were searched against a SwissProt human database (v. 56; 20333 sequences), using an in-house Mascot Deamon 2.2.2 server. Search criteria used were trypsin digestion, fix modification set as Methylthio (C), variable modifications set as N-terminal acetylation of protein, oxidation (M), iTRAQ 4plex at K and N-term of peptides, and allowing up to 1 missed cleavage, mass accuracy of 10 ppm on the parent ion and 0.50 Da on the fragment ions. This result dataset was then subjected to further statistical analysis using the Trans-Proteomic Pipeline (TPP, http://tools.proteomecenter.org/TPP.php), with PeptideProphet and ProteinProphet probability cutoffs set at 0.95 and 0.95, respectively, which filtered the reported protein hit dataset to an estimated false positive error rate of 0.7%, at an 87.1% sensitivity.

**Experimental Design and Sample Preparation**

We used an iTRAQ labeling-based protein identification and quantification approach with off-line basic reverse phase HPLC (bRP-HPLC) by Thermo LTQ-orbitrapTM mass spectrometer system to identify dysregulated proteins in gastric adenocarcinoma (GC) tumor cells and adjacent non-tumor mucosa captured by laser capture microdissection (LCM).

The tumor cells of the primary GCs and their adjacent non-tumorous mucosa were dissected by LCM and used for protein extraction by RapiGestTM lysis buffer. The quality and quantity of proteins extracted from the six pairs of microdissected samples (N1/T1 to N6/T6) were equally examined by SDS-PAGE followed by silver staining. The protein profiles of the six pairs are not similar to each other, indicating the heterogeneous nature of the patient sample pairs. Equal amounts of extracted proteins were then trypsin-digested following cysteine reduction and alkylation. Subsequently, peptides underwent second trypsin digestion and were resolved by SDS-PAGE following silver staining. After two step trypsin digestion, the tryptic peptides were labeled with iTRAQ buffer solution (114: non-tumor 1/3/5, 115: GC tumor 1/3/5, 116: non-tumor 2/4/6, 117: GC tumor 2/4/6).

Protein Identification by Two-dimensional Separation following LTQ-orbitrap MS analysis. Each iTRAQ-labeled peptides were pooled and fractionated by two-dimention HPLC system. The first dimension contained equally mixed iTRAQ labeled peptides separated by basic reverse phase chromatography into 40 fractions. The number of identified proteins is nearly equal from fraction 2 to fraction 38, indicating the separated efficiency of bRP-HPLC is desirable. Each fraction was dried at vaccum and resuspended in 0.3% formic acid for the following LC-MS/MS analysis.

**Identification of Differentially Expressed Proteins in GC Tissue Specimens**

The criteria of protein identification used by the protein and peptide prophet algorithm are protein prophet probability≧0.99, peptide prophet probability≧0.95, and at least two unique peptides are identified (0.99_2_0.95). Relative protein abundances were determined using the intensity of 114.1, 115.1, 116.1 and 117.1 reporter ions in the MS/MS scans of the iTRAQ-labeled peptides by libra algorithm.

Following the 0.99_2_0.95 criteria, 1559, 2765, and 2429 proteins are quantified. The candidates can be separated into up and down regulation group by dividing Tumor by Non-tumor (T/N ratio) using 1.5 and 0.67 fold as the cut-off value. The repeated presented protein candidates between each experiment were shown in Additional file 3: Table S2.

(A)


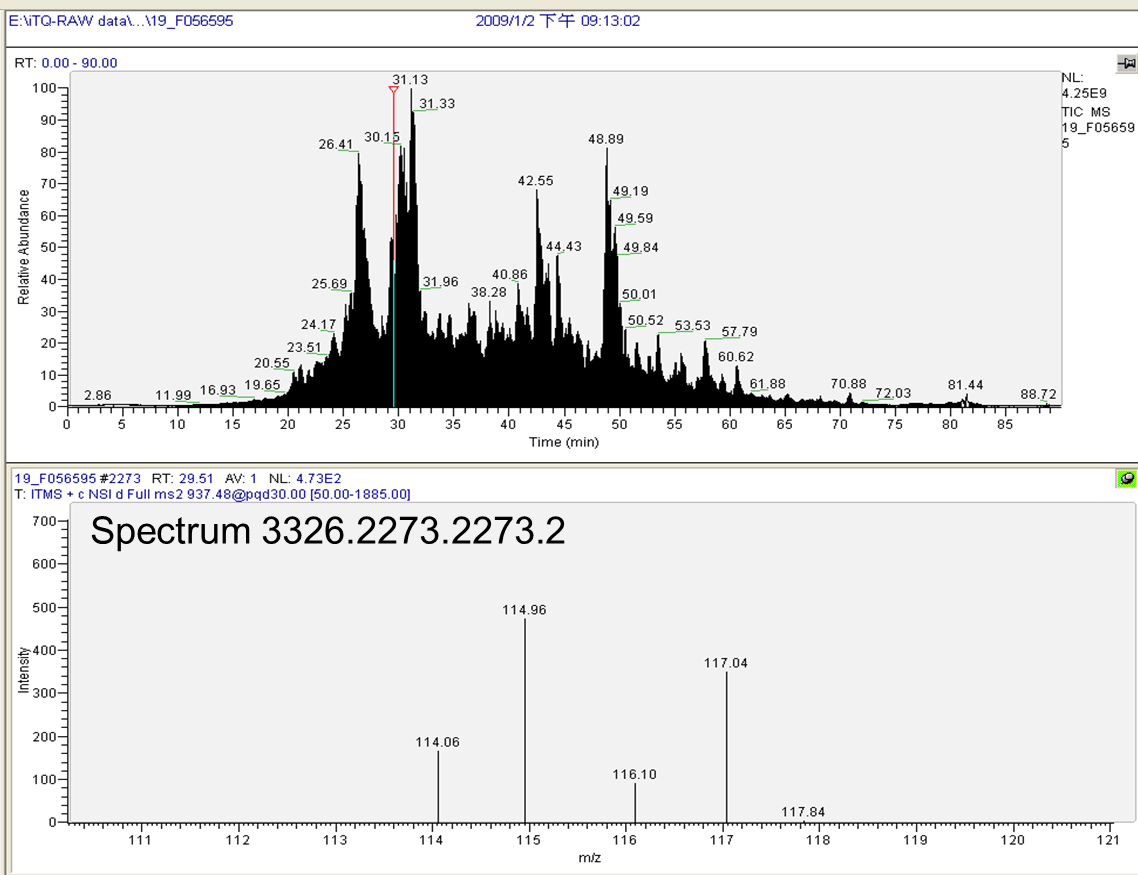


(B)


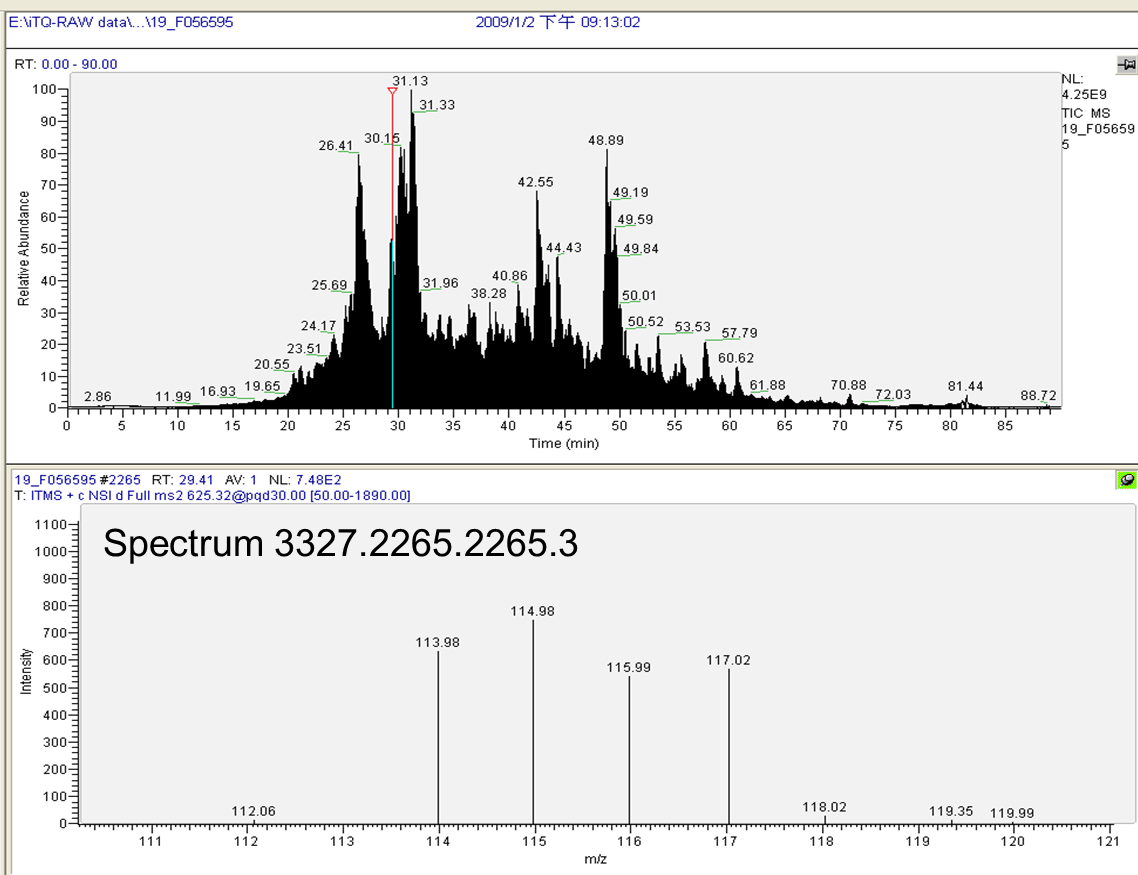


(C)


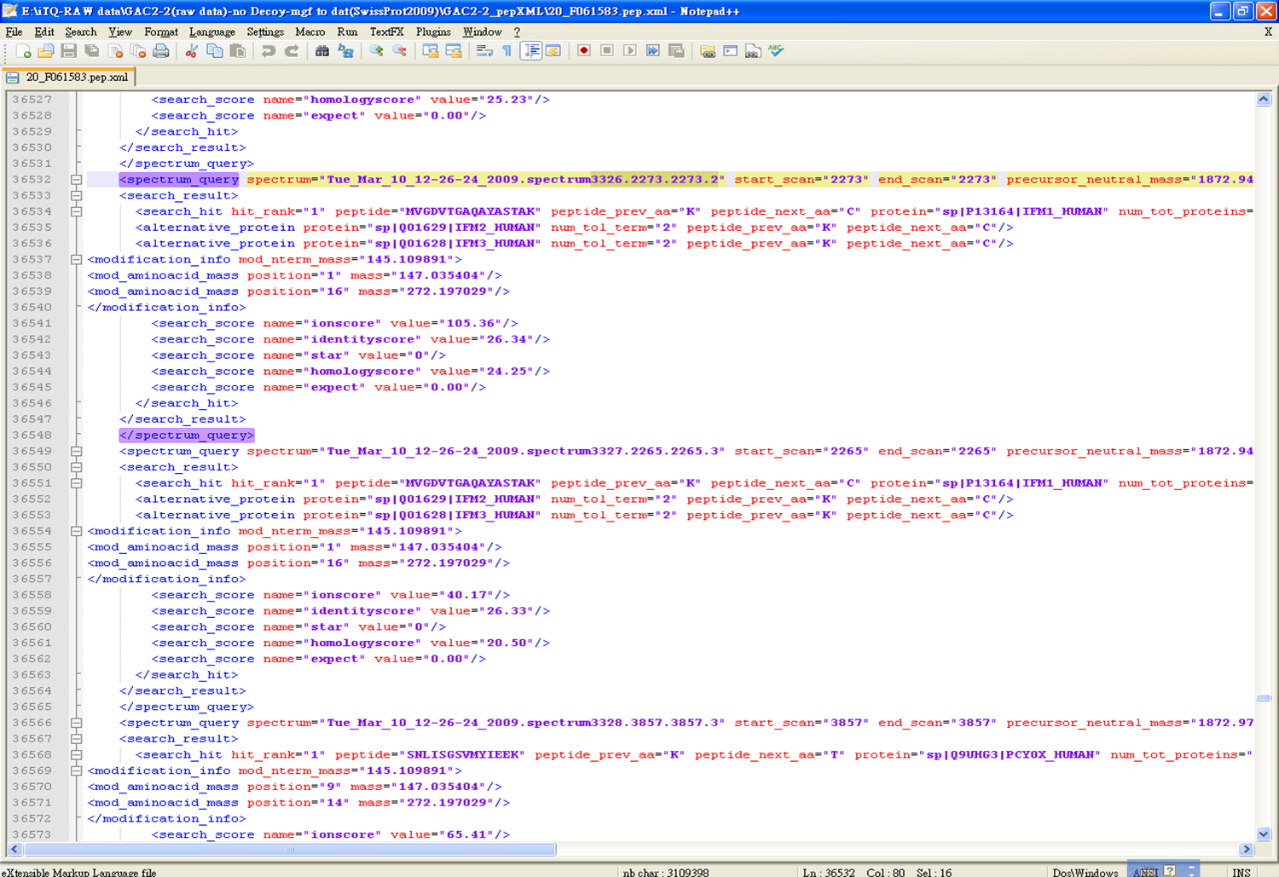


Images shows (A.B) MS spectra, MS/MS spectra, quantified iTRAQ reporter ions (114: non-tumor 3, 115: GC tumor 3, 116: non-tumor 4, 117: GC tumor 4) and (C) identified peptide sequences of peptide MVGDVTGAQAYASTAK from IFITM3.

**
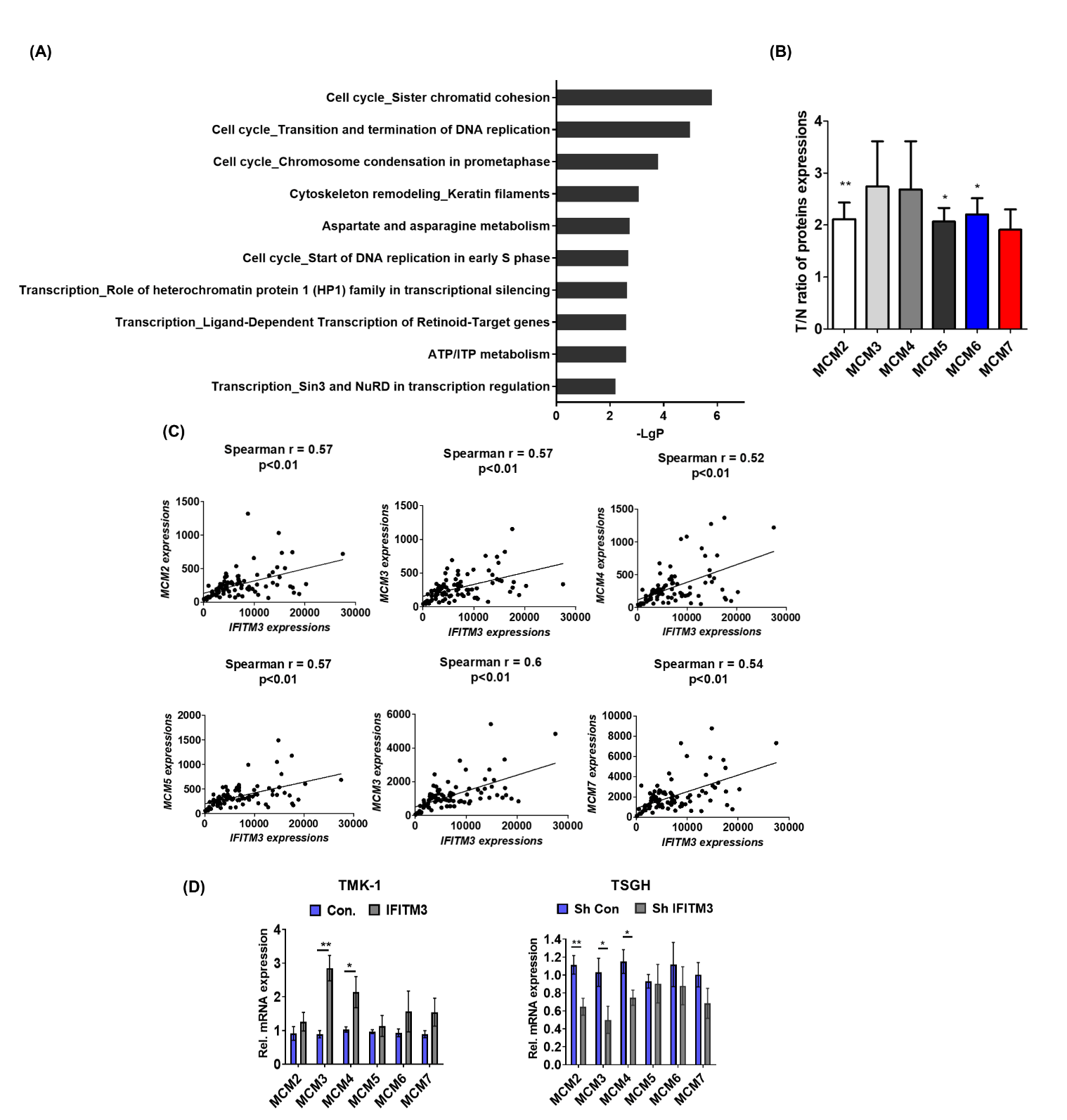
**

**Figure S1. MCM2-7 proteins are regulated by IFITM3 in GC proliferation**

(A) MetaCore analysis of 415 overexpressed proteins in GC tumors from our proteomic database. The signature genes involved in cell cycle regulation, metabolism and transcriptional regulation were shown. (B) Relative MCM2-7 proteins in GC tissues as compared to adjacent normal gastric tissues from our iTRAQ quantitative proteomic analysis (C) Spearman rank correlation coefficient of individual genes (including MCM2, MCM3, MCM4, MCM5, MCM6 and MCM7) against IFITM3 in 71 GC tissues from Cho Gastric dataset (GSE138861). (D) Quantitative RT-PCR analyses were conducted on MCM2-7 genes for their transcript expression in TMK-1 IFITM3-overexpression or TSGH IFITM3-depletion models (*p<0.05, **p<0.01).
